# Effectiveness of Exercise on Sleep Quality in Attention Deficit Hyperactivity Disorder: A Systematic Review and Meta-Analysis

**DOI:** 10.3390/children12020119

**Published:** 2025-01-22

**Authors:** Daniel González-Devesa, Miguel Adriano Sanchez-Lastra, Benito Outeda-Monteagudo, José Carlos Diz-Gómez, Carlos Ayán-Pérez

**Affiliations:** 1 Grupo de Investigación en Actividad Física, Educación, y Salud (GIAFES), Universidad Católica de Ávila, C/Canteros, 05005 Avila, Spain; 2 Well-Move Research Group, Galicia Sur Health Research Institute (IIS Galicia Sur), Servizo Galego de Saúde-Universidade de Vigo, 36310 Vigo, Spain; misanchez@uvigo.gal (M.A.S.-L.); jcdiz@uvigo.es (J.C.D.-G.); cayan@uvigo.es (C.A.-P.); 3 Departamento de Didácticas Especiáis, Universidade de Vigo, 36310 Vigo, Spain; 4 Facultad de Ciencias de la Educación y del Deporte, Universidade de Vigo, 36002 Pontevedra, Spain; beniouteda.166@gmail.com

**Keywords:** ADHD, physical activity, sleep duration, sleep status

## Abstract

**Objective**: This study aimed to systematically review the available evidence on the effects of exercise training programs on sleep quality in attention deficit hyperactivity disorder. **Methods**: Studies were searched in five electronic databases until March 2024. The methodological quality of the included studies was assessed using the Physiotherapy Evidence Database and Methodological Index for Non-Randomized Studies scales. **Results**: A total of five randomized clinical trials, two non-randomized comparative studies, and one single-arm trial were included. Self-reported sleep quality (*n* = 7) and objective sleep status (*n* = 3) were the main outcomes analyzed. Generally, exercise induced positive effects on self-reported sleep outcomes. The performed meta-analysis with data from 131 participants indicated that exercise showed a non-significant trend towards increasing objective sleep duration (Hedges’ g −2.67; 95% CI −11.33; 5.99, *p* = 0.185). While exercise appears safe for individuals with attention deficit hyperactivity disorder, its efficacy in managing sleep disturbances in this population remains uncertain. **Conclusions**: While there is evidence suggesting a positive impact of exercise on self-reported sleep quality, its efficacy for improving sleep duration could not be confirmed.

## 1. Introduction

Attention deficit hyperactivity disorder (ADHD) is a neurodevelopmental disorder that typically begins in childhood and is characterized by developmentally inappropriate and impairing levels of inattention, motor hyperactivity, and impulsivity. These difficulties often persist into adulthood [1]. 

People with ADHD often experience comorbidities that significantly impact their daily lives, with sleep problems being a prominent issue among them, with an estimated prevalence ranging from 23% to 73% in adolescents [2] and from 43% to 80% in adults [3], respectively. Sleep problems arise from various factors, including inadequate sleep hygiene, behavioral aspects of ADHD itself, side effects of medications used in ADHD treatment, and psychiatric or medical comorbidities [4]. Sleep problems can contribute to the onset or exacerbation of ADHD symptoms [5]. This bidirectional relationship underscores the importance of addressing sleep issues as part of the comprehensive management of ADHD.

Approaches for managing sleep problems in individuals with ADHD include both pharmacological and non-pharmacological strategies. While the efficacy of pharmacological treatment is considered controversial [6], non-pharmacological treatments stand out as an interesting alternative [7], with sleep hygiene measures being recommended as a first-line management approach for sleep disorders [8].

Sleep hygiene involves practicing strategies that have been proven to be effective in achieving more optimal sleeping patterns, such as engaging in exercise [9]. Exercise exerts positive effects on sleep through a multitude of pathways, engaging with circadian rhythm regulation, metabolic processes, immune function, thermoregulation, vascular health, mood regulation, and endocrine activity [10]. This is corroborated by scientific findings indicating that exercise not only enhances sleep quality but also reduces the time taken to fall asleep and improves subjective sleep experiences [11]. Moreover, the prescription of exercise has proven to be both viable and efficacious in managing sleep disturbances among affected individuals [12].

In this context, promoting exercise training among individuals with ADHD emerges as a promising strategy for addressing sleep problems. However, before offering guidance on exercise practice, psychologists, general practitioners, and sports physicians should have access to the most up-to-date evidence-based guidelines regarding its prescription and potential benefits for this population. Achieving this objective requires conducting systematic reviews that comprehensively synthesize and evaluate the existing scientific literature on this subject.

To the authors’ knowledge, no systematic review has been published that specifically explores the efficacy of exercise in managing sleep problems among individuals with ADHD. This study aims to determine whether exercise interventions improve sleep quality and duration in individuals with ADHD and to explore potential mediating factors.

## 2. Materials and Methods

This systematic review adhered to the guidelines established by the Preferred Reporting Items for Systematic Reviews and Meta-Analyses (PRISMA) [13]. The review protocol was formally registered with the Open Science Framework (OSF) and is accessible via the following link: https://doi.org/10.17605/OSF.IO/F53JK.

### 2.1. Search Strategy

A rigorous and systematic search was undertaken across five major electronic databases: MEDLINE/PubMed, Web of Science, EBSCOhost Environment Complete, Dialnet Plus, and Scopus. The search encompassed the entire publication history of each database, spanning from their inception to March 2024. To ensure exhaustive identification of relevant studies, a meticulously structured search strategy was employed, combining key terms and Boolean operators as follows: (“ADHD” OR “Attention Deficit Hyperactivity Disorder” OR “Hyperactivity”) AND (“Exercise” OR “Physical Activity” OR “Sport”) AND (“Sleep”). The Supplementary Materials Table S1 provides the detailed search strategy employed for each database.

### 2.2. Eligibility Criteria

An inclusive approach was adopted to ensure a comprehensive understanding of the available evidence on the research topic. Eligible studies for inclusion in this review consisted of peer-reviewed investigations that examined and reported on the effects of exercise interventions on sleep outcomes in individuals diagnosed with ADHD. Specifically, randomized controlled trials (RCTs) and non-RCTs were considered eligible provided that they met the following criteria: (a) they involved a structured exercise intervention performed by individuals with ADHD; (b) they used a pre-post design; (c) they included a group for comparison; and (d) they provided quantitative outcomes related to sleep parameters. Studies with mixed samples were excluded due to the lack of ADHD-specific data. Single-session exercise interventions were not analyzed as they do not constitute structured programs. Qualitative studies were omitted for lacking quantitative sleep data essential for systematic analysis. Finally, studies without full-text availability were excluded due to insufficient information for outcome evaluation.

### 2.3. Study Selection

The titles and abstracts of all identified studies were independently screened by two authors to assess their eligibility for inclusion. Following this preliminary review, the selected studies underwent a more detailed evaluation by both authors to confirm their suitability. Any disagreements or discrepancies encountered during this process were resolved through mutual discussion. In cases where uncertainty persisted regarding a study’s eligibility, a third author was consulted to provide additional input, and a consensus was achieved. Full-text copies of potentially relevant studies were obtained for further assessment. Additionally, the reference lists of the included articles, as well as studies citing them in Google Scholar, were examined to identify any additional eligible studies.

### 2.4. Data Extraction

Data were systematically extracted by one researcher from the original reports, including details on participants’ characteristics, intervention protocols, sleep-related outcomes, primary findings, adverse events, and drop-out rates. The extracted data were subsequently cross-verified by a second investigator to ensure accuracy. Whenever feasible, missing data were requested directly from the original study authors or marked as “NR” (Not Reported) when it was not possible to obtain them.

### 2.5. Quality Appraisal

The methodological quality of each RCT was evaluated using the Physiotherapy Evidence Database (PEDro) scoring system. For trials not indexed in the PEDro database, two authors independently assessed their quality following standardized criteria. Any discrepancies between the assessments were addressed through discussion and resolved by consensus. The quality of the included studies was classified based on the following thresholds: excellent (scores of 9–10), good (6–8), fair (4–5), and poor (<3) [14].

The methodological quality of comparative and single-arm studies was assessed by means of the Mixed Methods Appraisal Tool (MMAT) [15], which allows for the critical appraisal of different types of empirical designs. Two reviewers independently used the MMAT to critically evaluate the papers included in the systematic review. If any disagreement came up, they reevaluated the process until they reached an agreement. The MMAT consists of 7 questions, with the first 2 being screening questions that are common to all types of methodologies. The following 5 questions are specific to each of the 5 categories of study designs. Each question is answered in a ‘yes’, ‘can’t tell’, and ‘no’ format. Calculating an overall score is discouraged; instead, a color code rating is usually used (i.e., green for ‘yes’, yellow for ‘can’t tell’, and red for ‘no’) and a detailed presentation of the ratings is provided if necessary.

### 2.6. Statistical Analysis

The meta-analysis calculations were conducted using Microsoft Excel (version 2412) in combination with the Meta-Essentials Workbooks [16]. Effect sizes for quantitative dependent variables were calculated using Hedges’ g. A random-effects model was applied for all analyses to account for variability across studies, employing the inverse variance method.

To assess statistical heterogeneity and inconsistency, the I^2^ statistic was utilized. An I^2^ value of 0% indicates no observed heterogeneity, while higher values signify increasing levels of heterogeneity. In addition to calculating 95% confidence intervals (CIs), prediction intervals were determined to provide a more comprehensive representation of both the magnitude and consistency of the observed effects.

## 3. Results

### 3.1. Design and Samples

**Figure 1:**
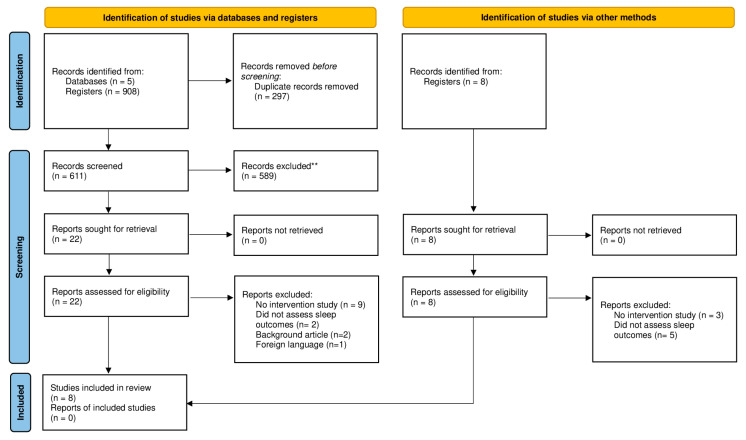
Flow diagram of the search and selection process for the inclusion of articles. ** Studies that were not related to the focus of the review were excluded.

Out of the 908 records initially obtained, a total of 8 studies, 5 RCTs [17,18,19,20,21], 2 non-randomized comparative investigations [22,23], and 1 single-arm trial [24] were finally analyzed (Figure 1). All the investigations were published between 2016 and 2023. A summary of their main characteristics is provided in Table 1.

The combined sample size across all reviewed studies amounted to 388 participants. The smallest study involved 14 participants [17], while the largest included 112 participants [20], respectively. The demographic characteristics of the participants varied significantly, with ages ranging from 6 to 54 years. Analysis of the data revealed that approximately 67% of the participants were male, representing a substantial majority. Six studies specifically examined children diagnosed with ADHD [18,19,20,22,23,24], one focused on adults [17], and another focused on undergraduates [21].

### 3.2. Interventions Characteristics

A diverse array of training programs was implemented, including jogging, Tai Chi, and Cooperative High-Intensity Interval Training, while other studies incorporated mixed exercise regimens (Table 1). The duration of exercise interventions ranged from 7 weeks [21] to 12 weeks [17,18,19,20,24]. Training frequencies varied between one session per week [18] and three sessions per week [17,19]. Session lengths ranged from 30 min [22,23] to 60 min [19,21,24]. Notably, three studies did not detail the methods used to monitor exercise intensity [18,21,24]. In the remaining studies, heart rate was used for this purpose.

### 3.3. Main Outcomes

#### 3.3.1. Self-Reported Sleep Quality

Seven of the included investigations assessed the effects of exercise on self-reported sleep quality [17,19,20,21,22,23,24].

According to the intra-group results, significant improvements in this outcome were observed in most of the experimental groups. However, Converse et al. [21] showed an increase in day-time dysfunction after 7 weeks of the Tai Chi program. Five studies assessed inter-group differences following the intervention, with three reporting significant improvements in the exercise groups compared to the control groups [17,22,23]. On the other hand, Liang et al. [19] and Geladé et al. [20] indicated no additional benefits of exercise over standard treatment protocols.

#### 3.3.2. Objective Sleep Status

Three studies utilized accelerometers to measure the objective sleep status as an outcome. Suarez-Manzano et al. [22] observed that exercise increased sleep time and reduced restlessness. In this line, Liu et al. [18] reported that exercise reduced sleep latency and time to wake after sleep onset and increased sleep efficiency. In both investigations, exercise was found to be more effective than usual treatments for decreasing time awake [22] and increasing total sleep duration, sleep latency, and sleep efficiency [18]. Notably, Liang et al. [19] did not observe any significant effect derived from exercising on objective sleep status.

**Figure 2:**
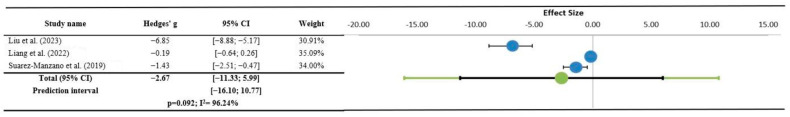
Forest plot on the effects of exercise on sleep duration [18,19,22].

The pooled data from three studies (*n* = 131) [18,19,22] indicated the absence of a significant impact of exercise on objective sleep duration (Hedges’ g −2.67; 95% CI −11.33; 5.99, *p* = 0.185), with high heterogeneity (I^2^ = 96.24%), as shown in Figure 2.

### 3.4. Dropouts and Adverse Events

A total of 85 dropouts were observed across the seven studies that provided information on this matter [17,19,20,21,22,23,24], with 36 of them occurring in the intervention group. The primary reasons for dropouts included participants voluntarily withdrawing, discontinuing the intervention, personal reasons, and incomplete data availability. None of the reviewed studies reported adverse effects derived from the training programs.

### 3.5. Methodological Quality

The methodological quality of the RCTs was considered fair in three studies [17,19,21] and good in two studies [18,20]. A full description of the quality analysis was also provided (see Table 2).

Non-randomized studies generally used appropriate measurements to assess outcomes and interventions, and interventions administered as intended. However, several weaknesses were noted. None of the three studies reviewed [22,23,24] controlled the confounders accounted for in design and analysis, included participants who were representative of the target population, or showed complete outcome data (Table 2).

## 4. Discussion

This study critically examined the scientific evidence on the effectiveness of exercise as a strategy for managing sleep problems in individuals with ADHD. To ensure a comprehensive analysis, the review extended beyond RCTs, considering several methodological factors. Firstly, it has been suggested that when the number of RCTs assessing non-pharmacological interventions is limited, drawing definitive conclusions becomes challenging. Including non-RCTs can thus provide a broader understanding of the evidence and help shape future research directions [25]. Secondly, non-RCTs contribute valuable insights when evaluating the feasibility of novel therapies, offering critical information on safety, adverse effects, and response rates [26]. Lastly, non-RCTs often deliver detailed information about intervention characteristics, such as exercise modality, duration, intensity, and any reported adverse effects. By broadening the scope of the review, this study captures a wider range of findings.

The reviewed literature encompassed a variety of exercise interventions, generally originating from studies with acceptable methodological quality. The findings from these studies contribute valuable insights into the potential role of exercise as a strategy for improving sleep hygiene in individuals with ADHD, enhancing our understanding of its benefits and practical application.

For instance, most of the studies assessed the efficacy of exercise on self-reported sleep quality. This is an important outcome measure given the high prevalence of sleep problems reported by parents of children with ADHD and adults with ADHD [27]. The investigations analyzed showed positive results in both children and adults, which is noteworthy given that pharmacological treatments may not always be effective for sleep problems in individuals with ADHD. Additionally, sleep medications may cause side effects or have interactions with other medications [28]. Initially, these results point out that exercise can be considered as a potential therapy for managing sleep problems among children with ADHD. These findings align with the positive effect of physical activity on sleep quality observed in children with autism spectrum disorder (ASD) [29], a neurological disorder that shares many similarities with ADHD [30]. It should also be taken into account that most of the studies proposed combined interventions that included exercises aimed at improving fitness levels, a marker of health that has been shown to be positively associated with sleep quality among children and adolescents [31]. However, it should not be overlooked that two of the reviewed investigations did not find exercise to have superior effects over the usual treatment. This aligns with findings from other studies, which have also reported results indicating that exercise may not have significant effects on certain self-reported sleep quality outcomes [9].

When studying sleep quality, it is recommended to use both subjective and objective methods for a comprehensive analysis [32]. In this regard, it has been reported that while subjective sleep problems are common in ADHD, further research is needed to objectively assess sleep alterations in this population [33]. In the present review, three studies provided objective sleep data, including sleep latency and sleep duration. Both outcomes are of interest since they have been found to differ significantly in both children and adults with ADHD [5]. Overall, the results were mixed; two studies indicated a positive effect of exercise on objective sleep, while one study found no significant impact. In this regard, scientific evidence is also controversial. For instance, among breast cancer patients, some authors argue that exercise was shown to be effective in improving subjective sleep problems, but no effect was observed on objective sleep measures [34]. However, other researchers have reported that exercise can positively affect objective sleep in this population [35]. Notably, although a systematic review and meta-analysis performed by Liang et al. [29] indicated that significant effects of exercise on objective sleep were mostly observed among children with ASD, not all investigations yielded positive results.

Previous investigations have suggested that children with ADHD have reduced sleep quantity [36]. In the present review, although the performed meta-analysis showed a positive trend toward the effects of exercise on sleep duration, the impact was not considered statistically significant. In children with TEA, the performance of physical activity was found to be significantly associated with sleep duration [37], while other studies focusing on healthy populations have also noted a lack of significant effect of exercise on this outcome [11]. Our findings suggest that exercise may not be as effective for increasing sleep duration in this population as pharmacological and behavioral interventions [7].

The mechanisms underlying the effect of physical exercise on sleep in individuals with ADHD remain unclear, though several possibilities have been proposed. Exercise may increase melatonin production due to the rise in body temperature it induces, aiding in sleep regulation [38]. Similarly, its benefits have been linked to melatonin secretion and its role in maintaining the circadian rhythm and sleep–wake cycle [18,19]. Another hypothesis is the thermoregulatory effect of exercise, where the body cools down more quickly afterward, promoting sleep induction [23,39]. Zhu et al. [39] also theorized that exercise optimizes vagus nerve function, which inhibits sleep induction, regulates cortisol production, and improves mood, fostering relaxation and better sleep. Additionally, reduced levels of brain-derived neurotrophic factor (BDNF) have been associated with sleep disturbances [40], and Klil-Drori and Hechtman [41] reported that aerobic exercise improved ADHD symptoms by increasing BDNF concentrations, suggesting a role for this protein in enhancing sleep in individuals with ADHD.

Regarding the efficacy of physical exercise for sleep problems compared to other therapies, Geladé et al. [20] found that the use of methylphenidate, neurofeedback, or physical exercise did not produce statistically significant improvements in any case. There is a lack of solid evidence regarding the effectiveness of pharmacological and non-pharmacological interventions in managing sleep problems in ADHD [7]. However, the same authors have highlighted the efficacy of pharmacological therapies (melatonin) and non-pharmacological therapies (cognitive-behavioral therapy), albeit with limited effects. In this context, Faraone et al. [42] present meta-analytic evidence indicating that stimulant drugs used to treat core ADHD symptoms worsen sleep quality in this population, as do non-stimulant drugs [43]. Therefore, pharmacological therapies should be administered cautiously. Overall, the efficacy of both types of therapies appears to be quite limited and even controversial in the case of pharmacological ones, leaving non-pharmacological options (cognitive-behavioral therapy, physical exercise, etc.) as a more viable alternative for addressing sleep problems in this condition. Ultimately, the literature consistently recommends multidisciplinary therapies (pharmacological and non-pharmacological) to maximize their effectiveness [43,44,45].

The analyzed studies included diverse exercise interventions in terms of content, intensity, weekly frequency, and participants’ age, resulting in significant heterogeneity that complicates the development of standardized exercise guidelines for individuals with ADHD. Despite this variability, the observed findings suggest that clinicians might recommend exercise interventions incorporating aerobic training combined with motor skill tasks aimed at improving coordination and agility for both adults and children. Interventions with a duration of 30–50 min per session, conducted 2–3 times per week, appeared to yield positive outcomes across various studies. Addressing this heterogeneity in future research by standardizing intervention protocols is essential to improve the consistency and reliability of exercise prescription guidelines.

Similarly, further studies are needed to confirm the impact of exercise as an adjunct therapy. Specifically, exploring the outcomes of combining exercise with proven effective approaches, such as cognitive-behavioral therapy, light therapy, or sleep hygiene, could provide valuable insights.

### Limitations

This appears to be the first systematic review and meta-analysis on the efficacy of exercise on sleep in individuals with ADHD. The obtained results lay the groundwork for future evidence-based guidelines on managing sleep disturbances in this population. However, there are some limitations that should be acknowledged for an accurate interpretation of the present findings. Firstly, the review included few studies, with most lacking details on ADHD type and severity, which limits the generalizability of the findings to diverse ADHD populations. Secondly, the meta-analysis combined data from three studies with heterogeneity and small sample sizes, resulting in low certainty of evidence and reducing the strength of the conclusions. Thirdly, the limited number of studies comparing different exercise programs made it difficult to determine the most effective regimen, further restricting the applicability of the results in clinical practice. Fourthly, the methodological quality of the studies was evaluated through the MMAT. While this tool is validated and widely used, its checklist is primarily based on generic criteria designed to assess and compare studies with diverse designs. As a result, it may not provide a comprehensive quality assessment for all the studies included in this review Finally, methodological issues, such as reliance on self-reports and cross-sectional designs, introduce potential bias, while the exclusion of grey literature and potential publication bias may have led to an overestimation of positive effects by omitting unpublished or negative findings. Consequently, these limitations weaken the overall robustness and generalizability of the findings.

## 5. Conclusions

The existing evidence regarding the prescription of exercise for managing sleep problems among individuals with ADHD remains uncertain. Preliminary data from this review suggests a positive effect of exercise on self-reported sleep quality, while its efficacy for improving sleep duration could not be confirmed. Given the small sample sizes, potential biases in self-reported data, and the significant heterogeneity in the exercise interventions studied, these conclusions should be considered tentative. These limitations make it difficult to generalize the results and establish definitive exercise prescription guidelines. As such, further research is needed to further determine whether exercise can be considered an optimal sleep hygiene behavior in this population.

The existing evidence regarding the use of exercise to manage sleep problems in individuals with ADHD remains inconclusive. Preliminary findings from this review suggest a potential positive effect of exercise on self-reported sleep quality; however, its efficacy in improving sleep duration could not be confirmed. Thus, there is still a need to determine whether exercise can be reliably recommended as an effective sleep hygiene practice for individuals with ADHD.

## Figures and Tables

**Table 1:** Overview of the descriptive characteristics of the included studies.

First Author (Year), Design and Country	Sample	Intervention	Outcomes	**Results**	**Dropouts and Adverse Events**
Svedell et al. (2023) [17] Design: RCT Country: Sweden	Participants (*n*): 14 adults with ADHD (EG: 9; CON: 5) Gender: EG: 3M + 6F; CON: 2M + 3F Age, years (mean; SD): EG: 33.7 ± 8.1; CON: 43 ± 12.6 BMI, kg/m^2^ (mean; SD): EG: 25.3 ± 5.1; CON: 29.1 ± 1.6 Duration of diagnosis, years: EG: 5.7 ± 3.3; CON: 3.4 ± 1.9 Medication, *yes/no*: EG: 5/4; CON: 3/2	Duration: 12 weeks EG Type: Mixed exercise program + treatment. Activities: Warm-up (6 min) light exercises; Cardio (6 min) cycling, running, jumping; Resistance training (23 min) lower body, upper body, core, balance; flexibility (10 min) stretching of large muscle groups; cool-down (5 min) light exercises. Volume: 50 min Frequency: 3 days/week Intensity: 60–90% HR_max_ CON Received treatment as usual	Self-reported sleep quality: ISI (score)	Intra-group *(p < 0.05)*—NR Inter-group *(p < 0.05*) > improvement in sleep quality in EG compared to CON (28.9 ± 16.7 vs. −51.4 ± 9.3%)	Dropouts: EG Drop out: *n* = 3 Participated in less than 60% of treatment sessions: *n* = 1 CON: Missing data: *n* = 1 Adverse events: NR
Liu et al. (2023) [18] Design: RCT Country: China	Participants (*n*): 33 children with ADHD (EG: 17; CON: 16) Gender: EG: 14M + 4F; CON: 12M + 4F Age, years (mean; SD): EG: 9.82 ± 1.24; CON: 10.44 ± 0.96 BMI, kg/m^2^ (mean; SD): EG: 18.87 ± 2.38; CON: 19.64 ± 2.78 Duration of diagnosis: NR Medication, *yes/no*: EG: 2/15; CON: 2/14	Duration: 12 weeks EG Type: Jogging Activities: Warm-up (10 min): The session began with a warm-up phase lasting 10 min. Jogging exercises (25 min): This segment included a variety of posture and motor learning exercises, such as skipping, high knees, and transitioning from hopping on two feet to one foot. These exercises were integrated into the jogging session to enhance motor skills. Cool-down (5 min): Light exercises were performed during the cool-down phase to gradually reduce physical exertion. Break (5 min) Volume: 45 min Frequency: 1 days/week Intensity: NR CON Received no exercise intervention and were asked to maintain their daily routine.	Self-reported sleep quality (Sleep Log): Sleep duration (h) Sleep latency (min) Sleep efficiency (%) Objective sleep status (Actigraphy Assessment): Sleep duration (h) Sleep efficiency (%) Wake after sleep onset (min)	Intra-group *(p < 0.05)* ↓ Sleep duration after intervention in CON (Actigraphy: 6.91 ± 0.22 vs. 5.75 ± 0.27 h) but ↑ when assessed with Sleep Log (7.14 ± 0.30 vs. 8.10 ± 0.24 h) ↓ Sleep latency after intervention in EG (Sleep Log: 45.36 ± 4.44 vs. 22.38 ± 2.7 min) ↑ Sleep efficiency after intervention in EG (Actigraphy: 82.64 ± 2.26 vs. 91.66 ± 2%; Sleep Log: 91.08 ± 0.84 vs. 95.64 ± 0.61%) ↓ Wake after sleep onset after intervention in EG (Actigraphy: 87.07 ± 11.20 vs. 39.30 ± 9.74 min) Inter-group *(p < 0.05*) > Sleep duration in EG compared to CON (Actigraphy: 6.79 ± 0.26 vs. 5.75 ± 0.27 h) < Sleep latency in EG compared to CON (Sleep Log: 22.38 ± 2.7 vs. 39.4 ± 2.26 min) > Sleep efficiency in EG compared to CON (Sleep Log: 95.64 ± 0.61 vs. 91.46 ± 0.86%)	Dropouts: NO Adverse events: NR
Liang et al. (2022) [19] Design: RCT Country: China	Participants (*n*): 80 children with ADHD (EG: 40; CON1: 40) + 40 children with typical development as health Control (CON2) Gender: EG: 30M + 10F; CON1: 32M + 8F Age, years (mean; SD): EG: 8.37 ± 1.42; CON1: 8.29 ± 1.27 BMI, kg/m^2^ (mean; SD): EG: 16.98 ± 3.14; CON1: 16.37 ± 2.83 Duration of diagnosis: NR Medication, *yes/no*: NR	Duration: 12 weeks + 12 weeks follow-up EG Type: Mixed exercise program Activities: Warm-up (10 min); aerobic exercise (20 min) rope skipping, cardio kickboxing and agility ladder; neurocognitive exercise (20 min) basketball, table tennis, and badminton; cool-down (10 min). Volume: 60 min Frequency: 3 days/week Intensity: 60–80% HR_max_ CON1 Received no exercise intervention and were asked to maintain their daily routine during the first 12 weeks. Thereafter, received intervention in the following 12 weeks. CON2 Received no exercise intervention and were asked to maintain their daily routine.	Self-reported sleep quality: • PSQI (score) ■ Sleep quality ■ Sleep latency ■ Sleep duration ■ Sleep efficiency ■ Sleep disturbance ■ Sleep medications ■ Day-time dysfunction ■ Total score Objective sleep status: Sleep duration (min) Sleep latency (min) Sleep efficiency (%)	Intra-group *(p < 0.05)* ↓ Sleep quality PSQI score after intervention in EG (0.95 ± 1.05 vs. 0.64 ± 0.81) ↓ Sleep latency PSQI score after intervention in EG (1.28 ± 1.10 vs. 0.90 ± 0.88) ↓ Sleep latency PSQI score after intervention in CON1 (1.33 ± 0.98 vs. 0.65 ± 0.71) ↓ Sleep disturbances PSQI score after intervention in EG (1.03 ± 0.49 vs. 1.03 ± 0.54) ↓ Total PSQI score after intervention in EG (6.08 ± 3.38 vs. 5.03 ± 2.28) ↓ Total PSQI score after intervention in CON1 (6.54 ± 3.03 vs. 4.18 ± 3.24) ↓ Sleep latency after intervention in CON1 (25.26 ± 21.40 vs. 16.97 ± 10.41 min) Inter-group *(p < 0.05*)—NO	Dropouts: EG Discontinued intervention: *n* = 1 Refused test: *n* = 2 CON: Discontinued intervention: *n* = 8 Lost control: *n* = 1 Adverse events: NR
Suarez-Manzano et al. (2021) [23] Design: Comparative Country: Spain	Participants (*n*): 80 children with ADHD. Final sample: 52 (EG: 28; CON: 24) Gender: EG: 11M + 17F; CON: 13M + 11F Age, years (mean; SD): EG: 10.92 ± 2.68; CON: 9.46 ± 2.54 BMI, kg/m^2^ (mean; SD): EG: 17.44 ± 2.18; CON: 17.06 ± 3.58 Duration of diagnosis: NR Medication, *yes/no*: NR	Duration: 10 weeks EG Type: C-HIIT program Activities: Warm-up (6 min); main (16 min) included a combination of cardiorespiratory, motor, and coordinative exercises carried out in pairs or groups (cooperative context), 4 sets ratio of 30:30 s; cool-down (8 min). Volume: 30 min Frequency: 2 days/week Intensity: 85–100% HR_max_ CON Asked not to change their usual physical activity habits, extracurricular activities, eating habits, or medication.	Self-reported sleep quality: PSQI (score)	Intra-group *(p < 0.05)* ↓ Total PSQI score after intervention in EG (7.86 ± 1.01 vs. 2.93 ± 1.76) ↓ Total PSQI score after intervention in CON (8.08 ± 0.93 vs. 6.88 ± 2.64) Inter-group *(p < 0.05*) < Total PSQI score in EG compared to CON after intervention (2.93 ± 1.76 vs. 6.88 ± 2.64)	Dropouts: No complete cognitive data (*n* = 16) Dropped out during the intervention or attended less than 75% of the sessions (*n* = 12) EG: *n* = 12 CG: *n* = 16 Adverse events: NR
Suarez-Manzano et al. (2019) [22] Design: Comparative Country: Spain	Participants (*n*): 30 children with ADHD Final sample: 20 children with ADHD (EG: 10; CON: 10) Gender: EG: 4M + 6F; CON: 6M + 4F Age, years (mean; SD): EG: 11 ± 0.94; CON: 10.7 ± 0.95 BMI, kg/m^2^ (mean; SD): EG: 17 ± 2.45; CON: 18.1 ± 2.28 Duration of diagnosis: NR Medication, *yes/no*: NR	Duration: 10 weeks EG Type: C-HIIT program Activities: Warm-up (10 min); main (16 min) included a combination of cardiorespiratory, motor, and coordinative exercises carried out in pairs or groups (cooperative context), 4 sets ratio of 30:30 s; cool-down (4 min). Volume: 30 min Frequency: 2 days/week Intensity: >80% HR_max_ CON Asked not to change their usual physical activity habits	Self-reported sleep quality: PSQI (score) Objective sleep status: Sleep duration (min) Time in bed (min) Times awake Times restless	Intra-group *(p < 0.05)* ↓ Total PSQI score after intervention in EG (7.4 ± 1.17 vs. 3.5 ± 1.08) ↑ Sleep duration after intervention in EG (280.1 ± 34.58 vs. 311.8 ± 24.51 min) ↓ Times awake after intervention in EG (1.7 ± 1.25 vs. 0.7 ± 0.68) ↑ Times awake after intervention in CON (1.1 ± 1.1 vs. 2.8 ± 1.99) ↓ Times restless after intervention in EG (17.2 ± 3.26 vs. 13.4 ± 4.29) ↑ Times restless after intervention in CON (11.4 ± 1.71 vs. 14.4 ± 4.06) Inter-group *(p < 0.05*) < Total PSQI score in EG compared to CON after intervention (8.5 ± 0.7 vs. 3.5 ± 1.08) < Times awake in EG compared to CON after intervention (0.7 ± 0.68 vs. 2.8 ± 1.99)	Dropouts: Incomplete data (*n* = 2) Did not reach the indicated intensity (*n* = 2) Attended less than 80% of the sessions (*n* = 6) EG: *n* = 5 CG: *n* = 5 Adverse events: NR
Geladé et al. (2017) [20] Design: RCT Country: The Netherlands	Participants (*n*): 112 children with ADHD Final sample: 92 (EG: 31; CON1: 33; CON2: 28) Gender: EG: 24M + 7F; CON1: 24M + 9F; CON2: 22M + 6F Age, years (mean; SD): EG: 9.55 ± 1.76; CON1: 9.81 ± 1.86; CON2: 8.97 ± 1.22 BMI: NR Duration of diagnosis: NR Medication, *yes/no*: EG: 14/17; CON1: 12/20; CON2: 21/7	Duration: 10–12 weeks + 6 months follow-up EG Type: Physical activity program Activities: Warm-up (5 min); followed by five 2 min moderate intensity exercises at 70–80% HR_max_. After a 5 min break, five 2 min vigorous intensity exercises at 80–100% HR_max_. Volume: 45 min Frequency: 30 sessions/total Intensity: Moderate–vigorous, 70–100% HR_max_ CON1 Type: Neurofeedback. Activities: Theta/beta training was applied with the aim of inhibiting theta activity (4–8 Hz) and reinforcing beta activity (13–20 Hz) at the vertex on a screen. Volume: 45 min Frequency: 30 sessions/total Intensity: NR CON2 Administration of medication, methylphenidate.	Self-reported sleep quality: Sleep Disturbance Scale (score)—Parent reports	Intra-group *(p < 0.05)*—NR Inter-group *(p < 0.05*)—NO	Dropouts: EG Discontinued intervention: *n* = 3 Lost at follow-up motivational reasons: *n* = 3 CON1: Discontinued intervention: *n* = 1 Lost at follow-up motivational reasons: *n* = 5 CON2: Discontinued intervention: *n* = 3 Medical contraindications: *n* = 2 Lost at follow-up motivational reasons: *n* = 5 Adverse events: NR
Converse et al. (2020) [21] Design: RCT Country: USA	Participants (*n*): 21 undergraduates with ADHD (EG: 9; CON1: 5; CON2: 7) Gender: 7M + 14F Age, years (mean; SD): 20.7 ± 1.5 BMI, kg/m^2^: NR Duration of diagnosis: NR Medication, *yes/no*: 16/5	Duration: 7 weeks Frequency: 2 days/week Volume: 60 min Intensity: NR EG Type: Tai Chi program Activities: Tai Chi principles followed by instruction in the 24-form Yang style sequence CON1 Active control (cardio-aerobic fitness) CON2 Inactive control (no contact)	Self-reported sleep quality: • PSQI (score) ■ Day-time dysfunction	↑ Day-time dysfunction PSQI score after intervention in EG (1.11 ± 0.60 vs. 1.78 ± 0.67) ↓ Day-time dysfunction PSQI score after intervention in CON1 (1.8 ± 1.3 vs. 1.50 ± 0.58) ↓ Day-time dysfunction PSQI score after intervention in CON2 (1.43 ± 0.98 vs. 1.2 ± 1.2)	Dropouts: CON1: Withdrew prior to intervention, “family emergency”: *n* = 1 CON2: Withdrew prior to intervention, “lack of time”: *n* = 1 Adverse events: NR
López-Sánchez et al. (2016) [24] Design: Single arm Country: Spain	Participants (*n*): 18 children with ADHD Gender: 18M Age, years (mean; SD): 10.05 ± 1.8; BMI, kg/m^2^: NR Duration of diagnosis: NR Medication, *yes/no*: NR	Duration: 12 weeks EG Type: After-school physical activity program Activities: Circuits and exercises aimed at improving their physical condition, especially muscle inhibition and postural control, with an emphasis on relaxation and self-esteem. Volume: 60 min Frequency: 2 days/week Intensity: Moderate to high	Self-reported sleep quality: PSQI (score)	↑ The number of good sleepers based on the PSQI score (cut-off point 5/21)	Dropouts: *n* = 6 Adverse events: NR

>: Greater; <: Lower; ↑: Increment; ↓: Decrement; ADHD: Attention Deficit Hyperactivity Disorder; BMI: Body Mass Index; C-HIIT: Cooperative-High Intensity Interval Training; CON: Control Group; HR: Heart rate; F: Female; EG: Experimental Group; ISI: Insomnia Severity Index; M: Male; NO: Not Observed; NR: Not Reported; PSQI: Pittsburgh Sleep Quality Index.

**Table 2:** Methodological quality appraisal of the included studies.

**Randomized Controlled Trials (PEDro Scale)**	**First Author, Year**				
	**Svedell et al. (2023)** [17]	**Liu et al. (2023)** [18]	**Liang et al. (2022)** [19]	**Converse et al. (2020)** [21]	**Geladé et al. (2017)** [20]
1. Random allocation	+	+	+	+	+
2. Concealed allocation	−	−	−	+	+
3. Baseline comparability	+	+	+	+	+
4. Blind subjects	−	−	−	−	−
5. Blind therapists	−	−	−	−	−
6. Blind assessors	+	−	−	+	−
7. Adequate follow-up	−	+	+	+	+
8. Intention-to-treat analysis	−	+	−	−	+
9. Between-group comparisons	+	+	+	−	+
10. Point estimates and variability	+	+	+	−	+
Total score	5/10	6/10	5/10	5/10	7/10
**Non-randomized trials (Mixed Methods Appraisal Tool, MMAT)**	**Suarez-Manzano et al. (2021)** [23]		**Suarez-Manzano et al. (2019)** [22]	**López-Sánchez et al. (2016)** [24]	
1. Participants representative of target population	−		−	−	
2. Appropriate measurements of outcome and intervention/exposure	+		+	−	
3. Complete outcome data	−		−	−	
4. Confounders accounted for in design and analysis	−		−	−	
5. Intervention administered (or exposure occurred) as intended	+		+	+	

## Data Availability

Data are contained within the article.

## Supplementary Materials

The following supporting information can be downloaded at: www.mdpi.com/article/10.3390/children12020119/s1. Table S1: Full search strategy for each database with arguments presented as they were used (30 March 2024).
