# Supplementary material for: Effectiveness of Exercise on Sleep Quality in Attention Deficit Hyperactivity Disorder: A Systematic Review and Meta-Analysis
Source: Children (Basel). 2025 Jan 22;12(2):119. doi: 10.3390/children12020119 (PMC11854823; doi:10.3390/children12020119)
Supplement: Supplementary file 1 [file children-12-00119-s001.zip › children-3435409-supplementary.pdf]

Supplementary Material S1. Full search strategy for each database with arguments presented as they were used (30 March 2024).

Data Base	Search strategy	Results
Web Of Science	TS=((“ADHD” OR “Attention Deficit Hyperactivity Disorder” OR “Hyperactivity”) AND (“Exercise” OR “Physical Activity” OR “Sport”) AND (“Sleep”))	343
Scopus	TITLE-ABS-KEY (“ADHD” OR “Attention Deficit Hyperactivity Disorder” OR “Hyperactivity”) AND (“Exercise” OR “Physical Activity” OR “Sport”) AND (“Sleep”)	399
EBSCOhost Environment Complete	(“ADHD” OR “Attention Deficit Hyperactivity Disorder” OR “Hyperactivity”) AND (“Exercise” OR “Physical Activity” OR “Sport”) AND (“Sleep”)	2
Dialnet Plus	(“ADHD” OR “Attention Deficit Hyperactivity Disorder” OR “Hyperactivity”) AND (“Exercise” OR “Physical Activity” OR “Sport”) AND (“Sleep”)	8
MEDLINE/PubMed	(“ADHD” OR “Attention Deficit Hyperactivity Disorder” OR “Hyperactivity”) AND (“Exercise” OR “Physical Activity” OR “Sport”) AND (“Sleep”)	156
	**TOTAL**	908
